# Supplementary material for: The Effects of BSA-Stabilized Selenium Nanoparticles and Sodium Selenite Supplementation on the Structure, Oxidative Stress Parameters and Selenium Redox Biology in Rat Placenta
Source: Int J Mol Sci. 2022 Oct 28;23(21):13068. doi: 10.3390/ijms232113068 (PMC9654536; doi:10.3390/ijms232113068)

The Effects of BSA-Stabilized Selenium Nanoparticles and Sodium Selenite Supplementation on the Structure, Oxidative Stress Parameters and Selenium Redox Biology in Rat Placenta

Milica Manojlović-Stojanoski[1], Slavica Borković-Mitić[1], Nataša Nestorović[1], Nataša Ristić[1], Svetlana Trifunović[1], Magdalena Stevanović[2], Nenad Filipović[2], Aleksandar Stojsavljević[3,4], Slađan Pavlović[1*]

[1] Institute for Biological Research "Siniša Stanković" – National Institute of the Republic of Serbia, University of Belgrade, Bulevar Despota Stefana 142, 11060 Belgrade, Serbia
[2] Group for Biomedical Engineering and Nanobiotechnology, Institute of Technical Sciences of the Serbian Academy of Sciences and Arts (SASA), Kneza Mihaila 35/IV, 11000 Belgrade, Serbia
[3] Faculty of Chemistry, University of Belgrade, Studentski trg 12-16, 11000 Belgrade, Serbia
[4] Innovative Centre of the Faculty of Chemistry, University of Belgrade, Studentski trg 12-16, 11000 Belgrade, Serbia
* Correspondence: sladjan@ibiss.bg.ac.rs

Supplementary material

Table S1. Chemical composition of rat diet.

**Chemical composition of rat diet**					
**General**		**Vitamins**		**Minerals**	
Proteins	min 20.00%	Vit A	min 12.000 U/kg	Zinc	min 30 mg/kg
Fat	min 5.00%	Vit D3	min 2.000 U/kg	Copper	min 15.00 mg/kg
Humidity	max 11.5%	Vit E	min 30.00 mg/kg	Iron	min 60 mg/kg
Cellulose	max 5.00%	Vit B1	min 4.00 mg/kg	Manganese	min 60 mg/kg
Ashes	max 8.00%	Vit B2	min 7.00 mg/kg	Iodine	min 0.15 mg/kg
Calcium	min 0.95%	Vit B6	min 6 mg/kg	Selenium	min 0.30 mg/kg
Phosphorus	min 0.70%	Vit B12	min 0.05 mg/kg		
Lysine	min 0.60%	Folic acid	min 1.0 mg/kg	**Antioxidants**	
Methionin	min 0.60%	Choline chloride	1.000.00 mg/kg	E-321, E-320, E-338, E202	min 125 mg/kg
Tryptophan	max 0.15%	Niacin	20.00 mg/kg		
		Pantothenic acid	min 10.00 mg/kg		

Figure S1. Body weight of gravid females on day 21 of pregnancy. IC-Intact controls; C-Controls; SeNPs-Selenium nanoparticles; NaSe-Sodium selenite. Significantly different: [A]SeNPs *vs* IC; [B]SeNPs vs C, P<0.05

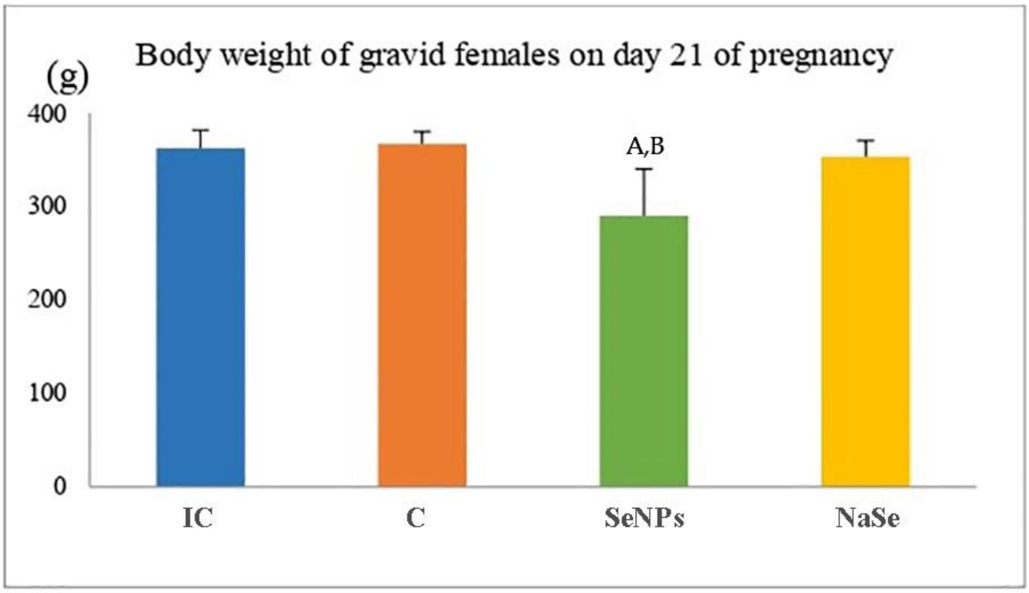

Figure S2. Absolut volume of the placenta and the volume density of placental layers in female (F) and male fetuses (M) on day 21 of pregnancy. IF-intact females; IM-Intact males; CF-Control females; CM-Control males; NF-Nanoselenium females; NM-Nanoselenium males; SF-Selenite females; SM-Selenite males.

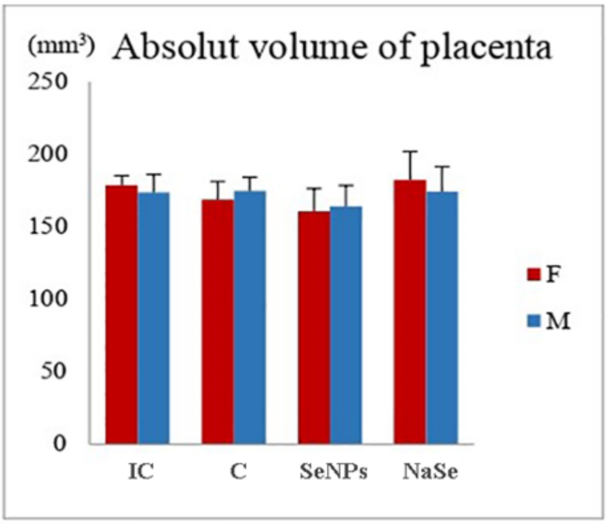

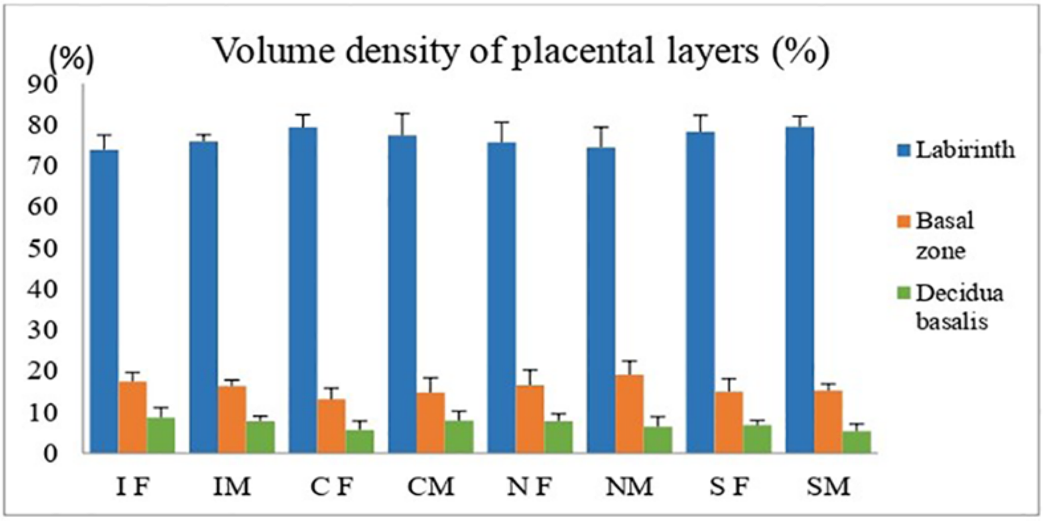

Figure S3. Synthesis and physicochemical characterization of SeNPs. A – Schematic presentation of the synthesis procedure with a macroscopic image of the obtained colloidal solution and lyophilized SeNPs. B, C – FTIR spectrum and XRD pattern of freeze-dried SeNPs, respectively. D, E – FE-SEM and TEM images of SeNPs, respectively.

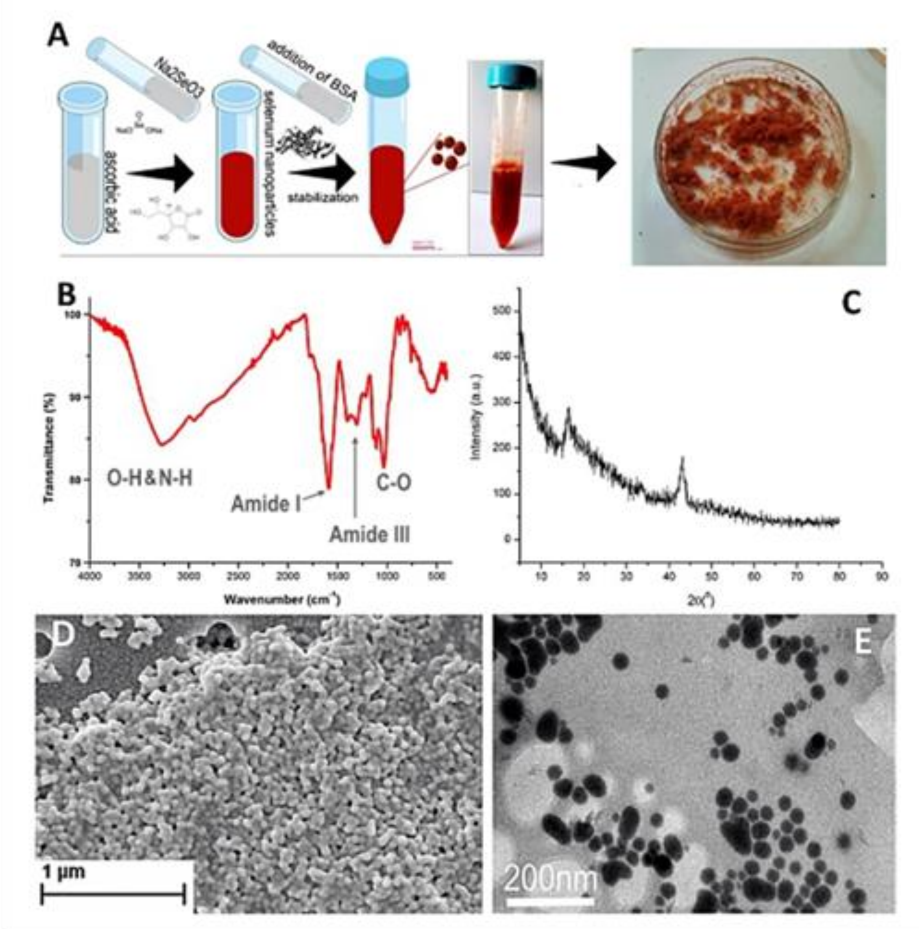

Supplement: Supplementary file 1 [file ijms-23-13068-s001.zip › ijms-1957767-supplementary.pdf]
